# IDentification of patients in need of general and specialised PALLiative care (ID-PALL©): item generation, content and face validity of a new interprofessional screening instrument

**DOI:** 10.1186/s12904-020-0522-6

**Published:** 2020-02-12

**Authors:** Fabienne Teike Lüthi, Mathieu Bernard, Michel Beauverd, Claudia Gamondi, Anne-Sylvie Ramelet, Gian Domenico Borasio

**Affiliations:** 1 Institute of Higher Education and Research in Healthcare, University of Lausanne and Lausanne University Hospital, Lausanne, Switzerland; 2 Palliative and Supportive Care Service, Lausanne University Hospital and University of Lausanne, Lausanne, Switzerland; 3 Palliative and Supportive Care Clinic, Oncology Institute of Southern Switzerland, Bellinzona, Switzerland

**Keywords:** Palliative care, Identification, Screening, Instrument, Item generation, Content validity, Interprofessional

## Abstract

**Background:**

Early identification of patients requiring palliative care is a major public health concern. A growing number of instruments exist to help professionals to identify these patients, however, thus far, none have been thoroughly assessed for criterion validity. In addition, no currently available instruments differentiate between patients in need of general vs. specialised palliative care, and most are primarily intended for use by physicians. This study aims to develop and rigorously validate a new interprofessional instrument allowing identification of patients in need of general vs specialised palliative care.

**Methods:**

The instrument development involved four steps: i) literature review to determine the concept to measure; ii) generation of a set of items; iii) review of the initial set of items by experts to establish the content validity; iv) administration of the items to a sample of the target population to establish face validity. We conducted a Delphi process with experts in palliative care to accomplish step 3 and sent a questionnaire to nurses and physicians non-specialised in palliative care to complete step 4. The study was conducted in the French and Italian-speaking regions of Switzerland. An interdisciplinary committee of clinical experts supervised all steps.

**Results:**

The literature review confirmed the necessity of distinguishing between general and specialised palliative care needs and of adapting clinical recommendations to these different needs. Thirty-six nurses and physicians participated in the Delphi process and 28 were involved in the face validity assessment. The Delphi process resulted in two lists: a 7-item list to identify patients in need of general PC and an 8-item list to identify specialised PC needs. The content and face validity were deemed to be acceptable by both the expert and target populations.

**Conclusion:**

This instrument makes a significant contribution to the identification of patients with palliative care needs as it has been designed to differentiate between general and specialised palliative care needs. Moreover, diagnostic data is not fundamental to the use of the instrument, thus facilitating its use by healthcare professionals other than physicians, in particular nurses. Internal and criterion validity assessments are ongoing and essential before wider dissemination of the instrument.

## Background

Palliative care has been clearly defined [1], but identifying individuals with palliative care needs is an ongoing challenge. In addition, the definition proposed by the World Health Organisation does not shed light on the distinction between needs for general and specialised PC. This distinction is, nevertheless, crucial to better understand the level of need in the population and thus improve access to appropriate PC and provide appropriate care [2–4]. Indeed, despite the development of PC, access to such care remains inequitable due to access to both specialists and generalists. This is particularly true for non-oncological patients and vulnerable populations [5]. Improved differentiation between patients requiring general and specialised PC will help the healthcare system to (i) better cope with the increase of PC needs related to demographic evolution, (ii) identify and meet the training needs of non-specialised PC professionals, and (iii) allow more efficient health spending through more equitable resource allocation and distribution [4–7]. As significant increases in PC needs are predicted, PC patient identification is an increasingly important public health concern. There is a consensus amongst experts in the field that PC should be initiated as early as possible in a patient’s disease trajectory, in order to provide best patient and relative centred care [8], for which effective needs identification is essential.

Proportions of patients with PC needs vary (9–73%), and people are often identified late in their disease trajectory, which bears important consequences for care [9–18]. These consequences include, but are not limited to (i) excess hospital mortality, whereby 80% of palliative patients die in hospital when the majority of people wish to die at home [19, 20]; (ii) suboptimal symptom management [21–23]; (iii) unplanned hospitalisations with long hospital stays [24, 25]; (iv) prescription of inappropriate treatments due to a lack of advance care planning [26, 27]; and, (v) insufficient support for the patient and their relatives [24, 28, 29]. On the other hand, when patients receive PC, their health and wellbeing is enhanced through improved pain relief, less dyspnea and depression, and enhanced quality of life, patient satisfaction and chances of dying at home [30–35]. Furthermore, psychological distress, decisional conflict, acute interventions or hospital readmissions are reduced [36–39], and quality of life for families is improved [40].

Existing instruments to facilitate early identification of patients in need of PC have significant limitations: (i) none of the existing instruments differentiate between patients in need of general versus specialised PC; (ii) most of the existing instruments have undergone limited psychometric testing; and (iii) available identification instruments require medical diagnostic criteria and have been primarily designed for use by physicians, therefore excluding other key healthcare professionals [41]. Interprofessional perspectives in PC are essential in order to provide holistic care for both patients and relatives throughout the disease trajectory until death. It is therefore crucial that professionals other than physicians are also involved from the beginning of the trajectory, especially at the identification stage. In addition, nurses generally have the closest contact with patients and relatives which facilitates the assessment of their needs. Indeed, in daily clinical practice, nurses often identify PC needs and try to relay them to physicians. A new instrument should help nurses to systematize their evaluation and provide them with the necessary tools for accurately reporting these needs to interprofessional teams.

## Methods

### Study design

The aim of this study is to describe the development of a new PC screening instrument to identify patients in need of general and specialised palliative care independently of their disease.

The development of this instrument was based on DeVellis et al. [42] and Streiner et al.’s [43] recommendations for scale development. The instrument was developed through the following four steps: [1] determine the concept to measure [2]; generate a set of items [3]; review the item set by experts (content validity) [4]; administer items to a sample of the target population (face validity).

The intended application of the instrument is to assist all healthcare professionals in evaluating patients’ palliative care needs. The target users of the instrument are mainly nurses and physicians, but potentially other healthcare professionals like social workers or physiotherapists. This instrument was designed for professionals, who do not necessarily have specific palliative care training, working in any acute care settings, except in intensive care units and emergency departments.

### Step 1: determine the concept to measure

A literature review was conducted to identify existing definitions of general and specialised palliative care to inform item generation. The Pubmed, CINHAL, Embase, Cochrane, JBI databases, Google Scholar, government and hospice websites were consulted between January and March 2016. Published studies and national/international recommendations were used to answer the research question: what are the currently available definitions for general PC and for specialised PC? All articles identified were collated and uploaded into EndNote X8/2016 (Clarivate Analytics, PA, USA). Titles and abstracts were then screened in order to keep only full texts related to the topic of interest. Definitions were extracted from the selected full text articles and collated in an Excel file for comparison.

### Step 2: generate a set of items

The aim of the second step was to obtain a list of possible items to include in the instrument. These items had to be relevant to the construct and the target population of interest, as well as for the context in which the instrument is intended to be used. This instrument is designed to be a clinician-reported outcome measure. As a result, the target population are non-specialised healthcare professionals and not patients. As the target population has some difficulties identifying patients requiring PC because professionals do not know the PC criteria, it was not deemed appropriate to include them at this stage of development.

For an instrument to meet the needs of busy healthcare professionals, it needs to be as concise and practical as possible. Regardless of whether the items referred to generalised or specialised PC, the first author (FTL) selected the relevant items from the literature, including published identification instruments [44–48]. This process was completed by a committee of interdisciplinary clinical experts in PC (CICE) including one clinical nurse specialist (FTL), one psychologist (MB1) and two physicians (MB2, GDB). This CICE was in charge of ensuring the relevance, comprehensiveness and comprehensibility for clinical practice, by analysing the data and the answers to the open questions, as well as by reformulating, adding or clustering items through discussions and deliberations [43]. An interprofessional working group was central in order to ensure that the different dimensions of PC are taken into account. The composition of the group was thought to be representative of the three professions most commonly involved in PC in Switzerland.

Following literature findings that people with life limiting non-oncological conditions have less access to palliative care [16, 23], the items were designed for patients with either oncological or non-oncological pathologies. Finally, the CICE determined which items belonged in the generalised PC or specialised PC categorisations.

### Step 3: review of initial set of items by experts

**Fig. 1:**
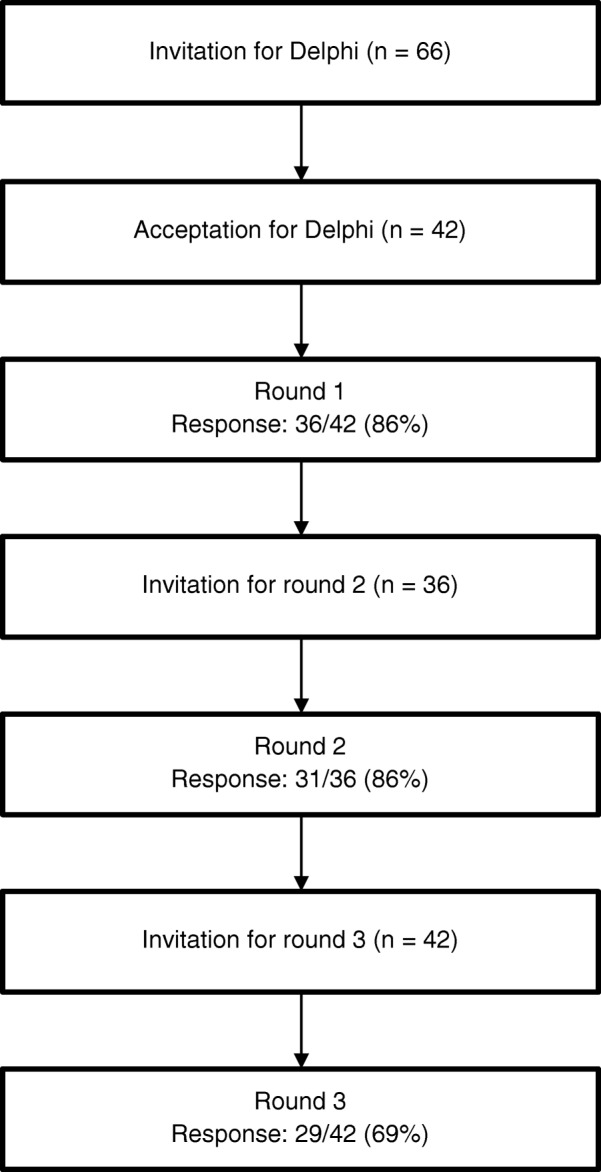
Delphi process flowchart with specialist palliative care healthcare professionals

A modified Delphi technique involving questionnaires with open and closed-ended questions was used to assess the relevance and the comprehensiveness of items related to general versus specialised PC from the final list generated in Step 2. These questionnaires were specifically developed for this participative process. Word documents were used to allow participants to report the items they chose for the instrument or to validate the final choices that were made. This occurred in the three rounds which allowed the content validity of the instrument to be established. The content validity can be enhanced during the development of an instrument through inclusion of experts of the discipline [49]. This method resulted in physicians and nurses from two linguistic regions of Switzerland (French and Italian), all experts in PC, to participating in the study [50–54]. A list of all nurses and physicians working in the different community-based specialist palliative care teams and all executive nurses and physicians of the PC units was compiled. Each of these professionals was contacted by email. This expert panel of clinicians specialised in PC was formed according to the following criteria: (i) a minimum of 3 years experience in PC, (ii) working in a hospice or in a hospital/community PC team, (iii) working in the French or Italian speaking regions of Switzerland, and (iv) having a sufficient oral and written level of French (as this part of the research was conducted in French). In addition, nurses were required to have undertaken specialised PC training or be a clinical nurse specialist or a head nurse. Physicians were required to be at least senior residents. Reasons for non-participation were not asked. Three rounds were necessary to achieve consensus (Fig. 1). In each round, a questionnaire including a list of items to be ranked according to the level of importance and one or two open questions for comments was sent to participants (all questionnaires are provided in Additional files 1, 2, 3). We carried out a pre-test with two nurses and one physician specialised in PC to assess the time necessary to complete questionnaires and the clarity of instructions.

In round 1, the participants ranked five items in order of priority for each set (general and specialised PC), with scores ranging from 5 (the most important) to 1 (the least important). The CICE recommended keeping items with a mean of ≥3.5 and selected by more than half of the participants. Each comment was reviewed for its pertinence by the CICE and through consensus was reworded when judged appropriate. Thus, the choice of items was influenced by the overall consistency of the item selection and by the pertinence of the comments. If an item pertaining to a specific issue was chosen in the first round, other items related to this issue were excluded from the second round. The same was true for items that had not been chosen at all in the first round.

In round 2, the same participants were asked to choose the items they believed should be retained for the instrument from the list of items remaining from the first round. As none of the items were chosen by more than half of the participants and reached a mean of ≥3.5, the CICE decided that only one of these two conditions had to be met.

In round 3, the aim was to confirm the item classification into either general or specialised PC and to ensure that the formulation was clear and understandable. A larger sample of experts, which also included those who participated in Round 1 and 2, were asked four yes/no questions to confirm (i) the relevance of the item selection in order to identify general and specialised PC patients, (ii) the comprehensibility and the clarity of the items and finally (iii) the name of the instrument. Two final sets of items (generalised PC and specialised PC) were developed at the end of the third round.

### Step 4: administer items to a sample of the target population for face validity

The aim of this last step was to ensure the face validity of the final set of items with the target population of end-users, namely nurses and physicians, without specialised PC training and working in acute care settings at a Swiss university hospital. Inclusion criteria were: i) being a clinical nurse specialist or a senior resident physician; ii) working in internal medicine, surgery, oncology, cardiology, pulmonology or ambulatory setting; iii) having worked full time for at least 3 years in one of these units (or the part time equivalent of 3 years full time). An invitation to participate was sent to all the clinical nurse specialists of the hospital, as well as to senior residents collaborating with the hospital’s mobile PC team. We sent participants a questionnaire including seven yes/no questions, pertaining to relevance, comprehensibility and feasibility. Space for extended responses for clarification or suggestions for “no” answers and other general comments were included in the questionnaire.

Descriptive analyses were conducted using IBM SPSS statistics 25. Classification of the comments was made by the first author (FTL) and then presented to the CICE for discussion.

## Results

### Step 1: determine the concept to measure

The origin of the construct of palliative care was grounded in the WHO definition (see above). The identification of patients requiring PC involves the ability to recognise them at the right time of their illness trajectory – depending on the patients’ and relatives’ needs – in order to introduce a general approach to PC at the right time and to be aware of the appropriate time to refer to a specialised PC team [2, 3, 16, 55]. General PC concerns all patients who have a life-threatening disease or who are at the end of life without complex bio-psycho-socio-spiritual issues. These patients may be cared for by healthcare professionals with basic knowledge of PC [55, 56]. General PC includes breaking bad news, relief of pain and other symptoms, establishing goals of care, and supporting patients and relatives throughout the continuum of care [6, 47, 57]. General PC may concern up to 80% of the proportion of patients requiring PC [2]. Specialised PC is for patients whose clinical situations are complex and/or unstable, and associated with high level of suffering of the patients and/or their relatives [55, 56, 58]. Specialised PC and treatments should be provided by an interprofessional team, including physicians, nurses, psychologists, social workers, and spiritual assistants, who are specifically trained in PC [47, 57].

### Step 2: generate of a set of items

The first author (FTL) selected 41 items from the literature (Table 1), including 21 items from the three most commonly used instruments: the Gold Standard Framework (GSF) [44], the Supportive and Palliative Care Indicator Tool (SPICT) [45], and the Necesidades Paliativas (NECPAL) [59] and 17 from other instruments or expert recommendations. Three different *surprise questions* with various expected times of death: 12 months, 6 months or “during the next months, weeks and days” were also integrated [60, 61]. Items representative of physical decline (e.g. decreased ability to perform self-care or less mobility) were retained. To ensure usability for key healthcare professionals other than physicians, items related to medical indicators of specific pathologies (e.g. cancer stages or spirometric criteria) were not included. Out of these 41 items, 32 were considered most relevant and understandable by the CICE. The CICE completed this first set of 32 items with 11 items derived from their clinical expertise. Clear, short items were written with dichotomous “yes” or “no” response categories because it is difficult for professionals to give a reliable estimation of the degree of difficulties encountered by the patients [43].

**Table 1:** Item generation process

Development steps	Item selection	Results
Step 2: generate a set of items	Initial set 1 of items (first author)	**41 items**: 21 from GSF, SPICT and NECPAL© 17 from other instruments and expert recommendations 3 different ‘surprise’ questions
	Initial set 2 of items (CICE)	**43 items**: 32 items selected from the initial set 11 new items added from the CICE clinical experience
	Categorisation of second set of items (CICE)	**Two part instrument**: 25 items for general PC 18 items for specialised PC
Step 3: review of initial set of items	Delphi round 1 (expert panel)	**5 items**: 3 items for general PC 2 items for specialised PC
	Delphi round 2 (expert panel)	**13 items**: 7 items for general PC 6 items for specialised PC
	Delphi round 3 (expert panel)	**Two lists of items** 7 items for general PC 8 items for specialised PC

### Step 3: review of initial set of items by experts

At least one physician and/or nurse of each of the specialised palliative care units and hospital or community PC team from each of the two linguistic regions were identified. A total of 71 professionals were screened for inclusion in the study; 66 met all inclusion criteria and 42 consented to participate. In the first round 19 nurses and 17 physicians participated; in the second 18 and 13; and in the third 15 and 14, respectively. Most of the participants had a specific training in PC (78%), with an average of 13 years of PC clinical practice experience.

#### Round 1

For the general PC part, out of the 25 items, three items reached a mean of more than 3.5: (i) the 12-month surprise question (M = 4.2 (SD = 1.4); *n* = 28), (ii) having life-limiting disease (M = 4.0 (SD = 1.3); *n* = 29) and (iii) the patient or family member is seeking palliative care (M = 3.5 (SD = 1.3); *n* = 25). For the specialised PC part, out of the 18 items, two items were selected: (i) persistence of uncontrolled symptoms (M = 4.4 (SD = 0.6); *n* = 27) and (ii) difficulty in assessing symptoms (M = 3.8 (SD = 0.9); *n* = 18) (Table 2). In addition and following the comments’ review by the CICE, three additional items were added to the Round 2 list, providing of a list of 25 items. Items with a mean ≤ 2 and chosen by less than five experts were not included in round 2.

**Table 2:** Item selection

Items	Round 1 M ± SD; *N* = 36	Round 2 M ± SD; *N* = 31
*General PC*		
Surprise question 12 months	4.1 ± 1.4; *n* = 20	
Surprise question 6 months	5.0 ± 0.0; *n* = 3	
Surprise question months, weeks or days	4.2 ± 0.8; *n* = 5	
PC required by healthcare professional	3.2 ± 1.8; *n* = 6	4.3 ± 1.0; *n* = 7
PC required implicitly or explicitly by patients or relatives	3.5 ± 1.3; *n* = 19	
PC required by patients/relatives	3.3 ± 1.4; *n* = 6	
Advanced illness, unstable symptoms	3.8 ± 1.6; *n* = 5	
Advanced illness and/or diminishing response to aetiological treatments	4.3 ± 0.5; *n* = 8	
Disease that cannot be treated, vital prognosis is underway	4.0 ± 1.3; *n* = 16	
Decreased response to treatment	1.7 ± 0.6; n = 3	
At least one disturbing symptom	2.6 ± 0.9; *n* = 11	4.1 ± 0.8; *n* = 11
Decrease in general condition	3.2 ± 1.3; n = 6	4.8 ± 0.5; *n* = 4
Poor or deteriorating performance status	2.6 ± 0.8; n = 7	3.8 ± 0.8; *n* = 9
General functional decline	1.9 ± 0.8; *n* = 8	5.0 ± 0.0; n = 8
Functional markers of decline	2.5 ± 0.6; n = 4	
Dependency on others for most care needs	*n* = 0	
Nutritional markers of decline	1.8 ± 0.7; n = 8	4.0 ± 0.6; n = 4
Significant weight loss	2.0 ± 0.8; n = 4	
Interruption of any vital support measures	2.7 ± 0.8; n = 6	4.0 ± 0.8; n = 11
Other markers of severity and extreme fragility	2.0 ± 1.0; *n* = 12	3.7 ± 0.7; n = 9
Sentinel events	n = 0	
≥ 2 unscheduled hospitalizations	1.8 ± 1.0; n = 9	3.9 ± 0.8; n = 9
≥ 2 concomitant diseases	*n* = 0	
Significant co-morbidity	2.0 ± 1.1; n = 7	3.7 ± 0.9; n = 9
Emotional distress	1.8 ± 0.7; n = 8	3.7 ± 0.8; n = 6
*Specialised PC*		
Specific population	4.1 ± 1.1; n = 11	3.6 ± 1.6; n = 8
Rapidly evolving illness	3.2 ± 1.8; n = 5	3.8 ± 1.7; n = 8
Need for complex and intense continuing care	3.0 ± 1.5; n = 12	2.3 ± 1.0; n = 9
Persistent and distressing symptoms	4.4 ± 0.6; *n* = 27	
≥ 3 symptoms > 5 on ESAS	3.8 ± 1.5; n = 8	3.6 ± 1.5; *n* = 10
Uncontrolled pain	3.2 ± 1.2; n = 9	4.3 ± 0.9; *n* = 16
Difficulties in assessing symptoms	3.8 ± 0.9; *n* = 18	3.5 ± 1.1; *n* = 15
Severe psychological and/or existential distress	2.6 ± 1.0; n = 18	2.6 ± 1.2; *n* = 17
Request for assisted suicide or euthanasia	1.4 ± 1.1; n = 8	2.2 ± 1.5; n = 6
Psychosocial distress patient and/or relatives	2.5 ± 0.9; n = 17	3.0 ± 1.1; n = 11
Difficulties in integrating information about the disease and/or prognosis	1.0 ± 0.0; n = 1	
Lack or insufficient support from relatives	2.0 ± 1.0; *n* = 3	
Social vulnerability	2.0 ± 0.0; n = 1	
Accompanying the patient is difficult	1.4 ± 0.8; n = 7	
Difficulties in communicating about therapeutic/care objectives	1.8 ± 0.4; n = 6	2.0 ± 1.4; n = 8
Significant disagreement, uncertainty or conflict	2.5 ± 1.5; *n* = 21	2.8 ± 1.4; *n* = 22
Need for support and/or second opinion for current decision-making	1.9 ± 0.8; n = 9	
Difficulties in writing advanced directives	n = 0	
Specialised PC team required by healthcare professional		3.6 ± 1.3; n = 5
Palliative sedation envisaged		2.6 ± 1.3; *n* = 14
Need for respite for the relatives		1.3 ± 0.6; n = 3

#### Round 2

Considering the general PC part, seven items with a mean score of ≥3.7 were selected. In order to minimise the number of items, the items related to the notion of vulnerability/frailty were grouped to obtain four items. Regarding the specialised PC part, two items obtained means ≥3.5 and were chosen by more than half of the participants. Four others obtained either means ≥3.5 or were selected by more than half of the participants. These six items were kept for the specialised PC part (Tables 2 and 3).

**Table 3:** items kept for the instrument at each round

Items kept after first round	Items kept after second round
General PC	
Surprise question 12 months	PC required by healthcare professional
PC required implicitly or explicitly by patients or relatives	At least one distressing symptom
Disease that cannot be treated, vital prognosis is underway	General functional decline
	Interruption of any vital support measures
	Other markers of severity and extreme fragility
	≥ 2 unscheduled hospitalizations
	Significant co-morbidity
	Emotional distress
Specialised PC	
Persistent and distressing symptoms	Difficulties in assessing symptoms
	Severe psychological and/or existential distress
	Psychosocial distress patient and/or relatives
	Difficulties in communicating about therapeutic/care objectives
	Significant disagreement, uncertainty or conflict
	Specialised PC team required by healthcare professional
	Palliative sedation envisaged

#### Round 3

69% of participants (*n* = 20) thought that the general PC items would help healthcare professionals non-specialised in PC to identify patients in need of general PC, and 76% (*n* = 22) estimated that the specialised PC items would facilitate the identification of patients in need of specialised PC. Half of the participants suggested modifying some items for better understanding (e.g: to replace the term ‘caregiver’ by ‘professional’; to give examples of what are considered life-sustaining measures; to replace ‘life-threatening disease’ by ‘disease that limits life expectancy’). These suggestions were taken into account in the final set of items. Finally, the proposed name ID-PALL© for IDentification of the patient in need of PALLiative care was approved by 76% (n = 22) of the participants; no other names were suggested.

Following the review of the written comments, some items were reworded. The goal of developing a short, consistent and user-friendly clinical instrument justified this process. Due to their different disciplines and backgrounds, the CICE members did not always immediately agree; intensive discussions were sometimes necessary to reach consensus. Reflection about the goal of developing a short, consistent and practical clinical instrument was helpful to finally reach consensus.

### Step 4: administer items to a sample of the target population for face validity

Twenty nurses and eight physicians out of a total of 24 invited nurses and 24 invited physicians working in acute care setting at a Swiss university hospital participated in the face validation (57%). Twenty-three participants (82%) considered that the items would help them to identify patients in need of general PC and 24 (86%) those in need of specialised PC. Twenty-seven professionals (96%) thought that classification of items into general and specialised PC is needed to appropriately care for these patients. Twenty-two found the length and the presentation of the instrument appropriate and understood how to use it and how to interpret the results. On the other hand, 15 (54%) asked for the clarity of some items to be improved. The main requests were to have fewer and shorter items; to clarify some items from the general part that were considered not specific enough; to arrange some items differently; to modify some terminology considered too vague (e.g ‘uncomfortable symptoms’) and to highlight keywords in each item. Following these suggestions, the wording of the relevant items were modified. Finally, two lists of items were obtained: one including seven items to identify patients in need of general PC and the other including eight items to identify specialised PC needs. A yes/no response categories are provided for each item. If one item is positive for the first part, the patient can be considered as in need of general PC. Evidence based recommendations for practice are then proposed and professionals are invited to complete the second part of the instrument. If two items are positive, the patient can be considered as in need of specialised PC. We recommend that each patient hospitalized for 24 to 48 h should be screened with ID-PALL©.

## Discussion

To the best of our knowledge, this is the first study that aims to develop and validate a screening instrument to identify patients in need of general or specialised PC independently of disease type. From the literature, this distinction is crucial in order to identify the most suitable moment for implementing adequate PC [3, 16, 55]. A rigorous process for instrument development was followed to obtain items that could identify patients in need of generalised or specialised PC. Both the content and face validity process of the instrument were deemed to be acceptable within both the expert and the target populations. The name ID-PALL© was chosen for the instrument that will be created based on these two lists of items.

Concerning the content validity, the qualitative feedback from the experts was pertinent and these comments allowed consensus to be achieved about the reformulation of certain items. However, in some instances, it was unclear as to how some comments could be used to modify the items because each participant contributed individually without a global vision of the instrument development that only the CICE had. The CICE’s work was therefore crucial, requiring intensive discussions and adjustments in order to maintain global coherence with all the responses and to reach consensus on two lists of relevant items that cover the bio-psycho-social and spiritual dimensions of PC and which include the most important challenges facing health professionals. The interprofessionality of the group was a key element for these discussions. To maximise the utilisation of an innovation, such as a new instrument, several factors are important: (i) perceived benefits, (ii) compatibility with users’ values, (iii) ease of use, (iv) experiment before implementation and (v) quick visibility of benefits [62]. Considering that time constraints are also a significant barrier to the adoption of innovative practice [62], ID-PALL© was conceived to be brief and containing as few questions as necessary while still taking into account the key domains of PC and retaining content validity. This led the CICE to reduce the number of items early in the process, despite the fact that this is not generally recommended by experts [43]. Nevertheless, the results of the face validity phase showed that the instrument’s brevity was much appreciated. As this screening instrument was designed to support healthcare professionals in their clinical reflection, brief evidence based recommendations will be added for initial guidance whenever general or specialised PC needs are identified by the instrument [63–65].

The referral rates for specialised PC remain low among health providers in all care settings, because of the difficulties in the timely identification of these patients [16–18, 35, 40]. This also means that it is difficult to know whether patients in need of general PC receive care that is adapted to their particular situation [16, 18, 55]. Therefore, we carefully structured the two lists of items in such a way that it would be evident to professionals that patients with life limiting diseases should be evaluated for potentially unmet PC needs at an early stage, even if they don’t meet the requirements for specialised PC. The main strength of the instrument is its two-part form, for general and specialised PC and its brevity and therefore quick utilisation. Additionally, due to its design and the fact that this instrument is not reliant on diagnosis data, this instrument has the potential to be used by healthcare professionals other than physicians, in particular by nurses as they have a more continuous presence and have a more holistic knowledge of the patients.

### Strengths and limitations

Methodological strengths of this study include the diversity of the expert panel working in multiple PC settings, in different parts of Switzerland, with different practices, and also with varied PC backgrounds that reflect the diversity of the field. Another strength is the inclusion of the target population in the face validity process, thus reducing possible dissatisfaction of future users [43].

Imposing a limit on the number of items poses a potential limitation. However, experts and clinicians had the opportunity to add items, if they deemed it relevant, at all stages of the design. The Delphi process, based on the ranking of the five most important items, did not allow for the establishment of a Content Validity Index (CVI), which is recommended in the literature. We chose a local expert panel for maximum transferability in the subsequent validation study, and acknowledge that this may limit generalisation to other French-speaking regions. In addition, while the CICE was an interprofessional working group, the absence of chaplains and social workers may limit its representativeness of the different dimensions of PC, particularly concerning spiritual and social aspects. With respect to the face validity, cognitive debriefing during the completion of the instrument may have provided valuable insights into the ways in which the instrument is completed and the different factors taken into account when responding. Cognitive debriefing has thus been planned as part of the subsequent implementation study. A further limitation of this study is the absence of service user input into the CICE. Service user input will feature prominently in the pilot implementation studies of the new validated instrument. Finally, the face validity was only conducted with hospital healthcare professionals, thus validation in different care settings should be conducted in the future.

## Conclusions

These results represent the first step of the validation process of the newly developed ID-PALL© instrument. The methods used in the development have resulted in an instrument that is brief and tailored to the needs of all health professionals, nurses in particular, who are confronted with patients with a potential need for PC. This instrument should allow the distinction between patients requiring general vs. specialised PC, regardless of their pathology. We are currently analysing the criterion validity of this instrument in order to assess its sensitivity and the specificity compared with the evaluation of a specialised interprofessional PC team including both nurses and physicians. An implementation study is currently being designed for the wider implementation of this instrument into clinical practice.

## Supplementary information

**Additional file 1.** Delphi questionnaire round 1. This questionnaire was send to the participants to choose the most relevant items for the instrument and the best formulation for each item. This questionnaire was originally in French. We present a literal translation in this article.

**Additional file 2.** Delphi questionnaire round 2. This questionnaire was send to the participants to choose the most relevant items for the instrument and the best formulation for each item. This questionnaire was originally in French. We present a literal translation in this article.

**Additional file 3.** Delphi questionnaire round 3. This questionnaire was send to the participants to assess the relevance and the comprehensibility of the final items, and also the name of the instrument. This questionnaire was originally in French. We present a literal translation in this article.

## Data Availability

The datasets used and analysed during the current study are available from the corresponding author on request.

## Abbreviations

CICE: Committee of interdisciplinary clinical experts
CVI: Content validity index
GSF: Gold standard framework
ID-PALL©: IDentification of patients in need of general and specialised PALLiative care
M: Mean
NECPAL: Necesidades paliativas
PC: Palliative care
SD: Standard deviation
SPICT: Supportive and palliative care indicator tool
WHO: World Health Organization
