# Supplementary material for: IDentification of patients in need of general and specialised PALLiative care (ID-PALL©): item generation, content and face validity of a new interprofessional screening instrument
Source: BMC Palliat Care. 2020 Feb 12;19:19. doi: 10.1186/s12904-020-0522-6 (PMC7017473; doi:10.1186/s12904-020-0522-6)
Supplement: Supplementary file 1 — Additional file 1. Delphi questionnaire round 1. This questionnaire was send to the participants to choose the most relevant items for the instrument and the best formulation for each item. This questionnaire was originally in French. We present a literal translation in this article. [file 12904_2020_522_MOESM1_ESM.docx]

**Delphi questionnaire (round 1)**

1. From the following list of items, could you rank, in order of relevance, the 5 items that, from your point of view, would best support non specialised healthcare professionals to identify patients in need of "general" palliative care and list them in the table below?

| The 5 items that best identify patients in need of « general » palliative care, in order of relevance, are : | |
| --- | --- |
| 1 |  |
| 2 |  |
| 3 |  |
| 4 |  |
| 5 |  |

When an item is declined in several formulations (a, b, c), please choose **the** formulation that seems the most relevant to you.

1a. Would you be surprised if this patient died in the next 6 months? (the answer « no » ascertains a patient in need of PC)

1b. Would you be surprised if this patient died in the next 6 months? (likewise)

1c. Would you be surprised if this patient died in the next months, weeks or days? (likewise)

2. As healthcare professional, would you consider that this patient requires palliative care or treatment at the moment?

3a. The patient or their family asks either explicitly or implicitly for palliative care or uniquely comfort care. They hint at limiting curative therapies or refuse specific treatments that are seen as curative.

3b. The patient and/or their entourage ask for palliative care or withdrawing treatment

4a. Advanced illness, unstable worsening of complex symptoms (burden)

4b. Advanced illness and or diminishing response to etiological treatments

4c. A disease that cannot be treated according to the current state of knowledge. The vital prognosis is underway.

5. Decreased response to treatment and reversibility

6. Presence of at least one disturbing symptom without an immediate response to treatment

7a. Decrease in general condition (Barthel ≤ 25 or PPS ≤ 60%) and/or loss of two or more activities of daily living (Katz index) despite adequate management

7b. Poor or deteriorating performance status (the person remains in bed or in a chair more than 50% of their waking time), with limited reversibility

7c. General functional decline and increased need for support

7d. Functional markers of decline, at least one of the following, within the last 6 months: serious and established functional dependence, loss of 2 or more activities of daily living (ADLs) despite adequate therapeutic interventions, clinical perception of functional decline (sustained, intense/severe, progressive, irreversible) not related to concomitant conditions

7e. The patient is dependent on others for most care needs due to physical and/or mental health problems.

8a. Nutritional markers of decline, at least one of the following, in the last 6 months: Albumin < 25 g/l weight loss of over 10% that is not related to an acute decompensation episode, Clinical perception of nutritional decline (sustained, intense/severe, progressive, irreversible) not related to concomitant conditions

8b. Significant weight loss (5-10%) in the last 3-6 months and/or low body mass index

9. Any vital support measures (e. g. gastrostomy, tracheostomy, invasive ventilation, transplantation, catecholamine) are not initiated or are interrupted for lack of medical indication

10. Other markers of severity and extreme fragility, at least two of the following, in the last 6 months: persistent decubitus ulcers (stage III-IV), recurrent infections (>1), delirium, persistent dysphagia, falls (>2)

11. Sentinel events e. g. serious fall, transfer to nursing home

12. At least two unplanned hospitalisations in the last 6 months

13a. At least two concomitant diseases

13b. Significant co-morbidity, responsible for and predictive of mortality and/or morbidity (Charlson Comorbidity Index)

14. Presence of emotional distress with psychological symptoms

2. From the following list of items, could you rank in order of relevance the 5 items that, from your point of view, would best support non specialised healthcare professionals to identify patients in need of “specialised” PC, once they have already been identified as being in need of “general” PC and list them in the table below?

| The 5 items that best identify patients in need of « specialised » palliative care, in order of relevance, are | |
| --- | --- |
| 1 |  |
| 2 |  |
| 3 |  |
| 4 |  |
| 5 |  |

1. Specific population: patient at high risk of symptomatic, psychological and/or existential crisis (e. g. risk of bleeding, acute dyspnea, incidental pain, etc.) or active psychiatric illness (e. g. depression, anxiety disorders, schizophrenia, dependence, etc.) or presence of a significant physical/cognitive disability or polymedication and/or intolerance/drug allergy
2. Rapidly evolving disease
3. Need for complex and intense continuous care in institution or at home
4. Persistent and distressing symptoms refractory to usual treatment within 48 hours
5. Presence of 3 or more symptoms greater than 5 on the ESAS
6. Uncontrolled pain with first-line analgesics such as opioids and/or adjuvants
7. Difficulties in assessing physical, psychological, social or spiritual symptoms
8. Presence of severe psychological and/or existential distress, for example: wish to die, loss of sense/hope, feeling isolated, feeling of being a burden
9. Request for assisted suicide or euthanasia
10. Psychosocial distress of the patient and/or family with respect to disease progression, death or other related factors
11. Difficulty for the patient and/or family and friends in integrating information about the disease and/or prognosis
12. Lack or insufficient support from relatives or need for support for relatives
13. Social vulnerability of the patient or his or her family or problems related to the cultural context
14. Accompanying the patient is difficult for the primary health care professionals due to patient/relative defense mechanisms and/or family conflicts
15. Difficulties in communicating about therapeutic/care objectives in life-limiting illness
16. Significant disagreement, uncertainty or conflict among the patient, team and/or family regarding, for example, important decisions about medical treatment, choice of resuscitation code, ethical concerns or complex decision-making
17. Need for support and/or second opinion for current decision-making or in anticipation of suspension/non-initiation of futile treatments, and/or disagreement on the resuscitation code
18. Inability of the front-line team to assist the patient in writing advanced directives

**Complementary questions**

3. Is there any item(s) that has (have) not been proposed and that you feel is (are) essential? If yes, which one(s)? (specify whether the criteria are "general" or "specialised")

**4.** How long have you been working in a PC service or specialised PC team : years

**5.** Have you completed specialisation training in palliative care?

□ Yes □ No

If yes, which one : □ DAS in PC □ MAS in PC □ Other (precise) :

**6**. Do you have any other comments ?

**Thank you for your participation**
