# Supplementary material for: IDentification of patients in need of general and specialised PALLiative care (ID-PALL©): item generation, content and face validity of a new interprofessional screening instrument
Source: BMC Palliat Care. 2020 Feb 12;19:19. doi: 10.1186/s12904-020-0522-6 (PMC7017473; doi:10.1186/s12904-020-0522-6)
Supplement: Supplementary file 2 — Additional file 2. Delphi questionnaire round 2. This questionnaire was send to the participants to choose the most relevant items for the instrument and the best formulation for each item. This questionnaire was originally in French. We present a literal translation in this article. [file 12904_2020_522_MOESM2_ESM.docx]

**Delphi questionnaire (round 2)**

1. From the following list of items, please rank, in order of relevance, up to 3 items that, from your point of view, would best support non specialised healthcare professionals to identify patients in need of "general" palliative care and that are not included in the 3 items already selected. Thank you to list them in the table below.

| The 3 items that best identify patients in need of « general » palliative care, in addition to those that have already been selected and in order of relevance, are : | |
| --- | --- |
| 1 |  |
| 2 |  |
| 3 |  |

When an item has been declined in several formulations (a, b, c), please choose **the** formulation that seems the most relevant to you.

1. As healthcare professional, would you consider that this patient requires palliative care or treatment at the moment?

2. Presence of at least one distressing symptom without an immediate response to treatment

3a. Decrease in general condition (Barthel ≤ 25 or PPS ≤ 60%) and/or loss of two or more activities of daily living (Katz index) despite adequate management

3b. Poor or deteriorating performance status (the person remains in bed or in a chair more than 50% of their waking time), with limited reversibility

3c. General functional decline and increased need for support

4. Nutritional markers of decline, at least one of the following, in the last 6 months: Albumin < 25 g/l weight loss of over 10% that is not related to an acute decompensation episode, Clinical perception of nutritional decline (sustained, intense/severe, progressive, irreversible) not related to concomitant conditions

5. Any vital support measures (e. g. gastrostomy, tracheostomy, invasive ventilation, transplantation, catecholamine) are not initiated or are interrupted due to lack of medical indication

6. Other markers of severity and extreme fragility, at least two of the following, in the last 6 months: persistent decubitus ulcers (stage III-IV), recurrent infections (>1), delirium, persistent dysphagia, falls (>2)

7. At least two unplanned hospitalisations in the last 6 months

8. Co-morbidité importante, responsable et prédictive de la mortalité et/ou de la morbidité (Charlson Comorbidity Index) (significant comorbidity, responsable for mortality and or modridity)

9. Presence of emotional distress with psychological symptoms

2. From the following list of items, could you rank in order of relevance up to 5 items that, from your point of view, would best support non-pecialised healthcare professionals to identify patients in need of “specialised” PC, once they were already identify in need of “general” PC, in addition to the 2 items that have already been selected. Please list them in the table below.

| The 5 items that best identify patients in need of « specialised » palliative care, in order of relevance, are | |
| --- | --- |
| 1 |  |
| 2 |  |
| 3 |  |
| 4 |  |
| 5 |  |

1. Specific population: patient at high risk of symptomatic, psychological and/or existential crisis (e. g. risk of bleeding, acute dyspnea, incidental pain, etc.) or active psychiatric illness (e. g. depression, anxiety disorders, schizophrenia, dependence, etc.) or presence of a significant physical/cognitive disability or polymedication and/or intolerance/drug allergy

2. Rapidly evolving disease

3. Need for complex and intense continuing care in institution or at home

4. Presence of 3 or more symptoms greater than 5 on the ESAS

5. Uncontrolled pain with first-line analgesics such as opioids and/or adjuvants

6. Difficulties in assessing physical, psychological, social or spiritual symptoms

7. Presence of severe psychological and/or existential distress, for example: wish to die, loss of sense/hope, feeling isolated, feeling of being a burden

8. Request for assisted suicide or euthanasia

9. Psychosocial distress of the patient and/or family with respect to disease progression, death or other related factors

10. Difficulties in communicating about therapeutic/care objectives in life-limiting illness

11. Significant disagreement, uncertainty or conflict among the patient, team and/or family regarding, for example, important decisions about medical treatment, choice of resuscitation code, ethical concerns or complex decision-making

12. As a healthcare professional do you consider that this patient requires a specialized palliative care team (mobile team or palliative care unit)?

13. Palliative sedation envisaged

14. Need for respite for the relatives who have reached their limits

**Complementary questions**

**3.** Is the wording of the following item acceptable to you, and in particular the formal inclusion of "implicit or explicit" in the statement?

The patient or their family asks either explicitly or implicitly for palliative care or uniquely for comfort care. They hint at limiting curative therapies or refuse specific treatments that are seen as curative.

□ Yes □ No

If not, what do you propose ?

**Thank you for your participation**
