# Supplementary material for: IDentification of patients in need of general and specialised PALLiative care (ID-PALL©): item generation, content and face validity of a new interprofessional screening instrument
Source: BMC Palliat Care. 2020 Feb 12;19:19. doi: 10.1186/s12904-020-0522-6 (PMC7017473; doi:10.1186/s12904-020-0522-6)
Supplement: Supplementary file 3 — Additional file 3. Delphi questionnaire round 3. This questionnaire was send to the participants to assess the relevance and the comprehensibility of the final items, and also the name of the instrument. This questionnaire was originally in French. We present a literal translation in this article. [file 12904_2020_522_MOESM3_ESM.docx]

**Delphi questionnaire (round 3)**

1. In your opinion, do all the items selected for the instrument “identification of patient in need of general or specialised palliative care” allow identification of all patients in need of general PC?

□ Yes □ No

If not, which item(s) could rectify this?

2. In your point of view, do all the items selected for the instrument “identification of patient in need of general or specialised palliative care” allow identification of all patients in need of specialised PC?

□ Yes □ No

If not, which item(s) could rectify this?

3. Are there any items that you would re-word?

□ Yes □ No

If yes, which one(s) and how ?

4. Do you believe the name ID PALL is suitable for this instrument?

□ Yes □ No

If not, what do you suggest ?

5. Other comments

**Thank you for your participation**
